# Ultrafine particles from diesel vehicle emissions at different driving cycles induce differential vascular pro-inflammatory responses: Implication of chemical components and NF-κB signaling

**DOI:** 10.1186/1743-8977-7-6

**Published:** 2010-03-22

**Authors:** Rongsong Li, Zhi Ning, Rohit Majumdar, Jeffery Cui, Wakako Takabe, Nelson Jen, Constantinos Sioutas, Tzung Hsiai

**Affiliations:** 1 Biomedical Engineering and Cardiovascular Medicine, USC, Los Angeles, CA 90089, USA; 2 Civil and Environmental Engineering, USC, Los Angeles, CA 90089, USA

## Abstract

**Background:**

Epidemiological evidence supports the association between exposure to ambient particulate matter (PM) and cardiovascular diseases. Chronic exposure to ultrafine particles (UFP; *D*_p _<100 nm) is reported to promote atherosclerosis in ApoE knockout mice. Atherogenesis-prone factors induce endothelial dysfunction that contributes to the initiation and progression of atherosclerosis. We previously demonstrated that UFP induced oxidative stress via c-Jun N-terminal Kinases (JNK) activation in endothelial cells. In this study, we investigated pro-inflammatory responses of human aortic endothelial cells (HAEC) exposed to UFP emitted from a diesel truck under an idling mode (UFP1) and an urban dynamometer driving schedule (UFP2), respectively. We hypothesize that UFP1 and UFP2 with distinct chemical compositions induce differential pro-inflammatory responses in endothelial cells.

**Results:**

UFP2 contained a higher level of redox active organic compounds and metals on a per PM mass basis than UFP1. While both UFP1 and UFP2 induced superoxide production and up-regulated stress response genes such as heme oxygenease-1 (HO-1), OKL38, and tissue factor (TF), only UFP2 induced the expression of pro-inflammatory genes such as IL-8 (2.8 ± 0.3-fold), MCP-1 (3.9 ± 0.4-fold), and VCAM (6.5 ± 1.1-fold) (n = 3, *P *< 0.05). UFP2-exposed HAEC also bound to a higher number of monocytes than UFP1-exposed HAEC (Control = 70 ± 7.5, UFP1 = 106.7 ± 12.5, UFP2 = 137.0 ± 8.0, n = 3, *P *< 0.05). Adenovirus NF-κB Luciferase reporter assays revealed that UFP2, but not UFP1, significantly induced NF-κB activities. NF-κB inhibitor, CAY10512, significantly abrogated UFP2-induced pro-inflammatory gene expression and monocyte binding.

**Conclusion:**

While UFP1 induced higher level of oxidative stress and stress response gene expression, only UFP2, with higher levels of redox active organic compounds and metals, induced pro-inflammatory responses via NF-κB signaling. Thus, UFP with distinct chemical compositions caused differential response patterns in endothelial cells.

## Background

Exposure to atmospheric particulate matter (PM) is associated with cardiovascular and respiratory diseases [1,2]. Diesel and gasoline vehicle emissions in the urban areas have dominant contributions to ambient particles, especially those in the ultrafine range (*D*_p _< 100 nm). Because of their small size and large surface area per unit mass, ultrafine particles (UFP) have demonstrated unique biochemical characteristics, such as enhanced ability to adsorb or absorb organic molecules and to penetrate cellular targets in the human pulmonary and cardiovascular system [3-5]. Inhaled nano-sized particles in air pollutant can transmigrate across human pulmonary epithelium into systemic arterial circulation [6-8]. Circulating nano-sized particles may deposit at so-called hot spots such as artery bifurcations and accumulated to high concentration[9]. Recent studies suggest that UFP may be directly transported to the cardiac vasculature where they induced arrhythmias, reduced myocyte contractility, and decreased coronary blood flow [10,11]. Studies by Brook *et. al*. demonstrated that UFP from air pollution raise blood pressure and impair vascular function [12-14]. Araujo *et al*. reported that UFP promote atherosclerosis in ApoE-null mice [15]. However, the mechanism(s) by which UFP cause adverse cardiovascular effects remain largely unknown.

Generation of reactive oxygen species and activation of pro-inflammatory pathways have been implicated in the toxicity of inhaled particles. Both animal and human studies support the notion that vascular oxidative stress is closely linked to UFP-associated cardiovascular diseases [16-18], and that c-Jun N-terminal kinases (JNK) signal pathway plays an important role in UFP-induced oxidative stress[19,20]. While fine particles (PM_2.5_; *D*_p _< 2.5 μm) induced IL-6 and IL-8 expression in monocytes, diesel exhaust particles (DEP) induced inflammatory responses in epithelial cells [21-23] and macrophages [24] present in human airways. Exposure to UFP further increased the expression of inflammatory genes such as MCP-1, IL-6 and IL-8 in human pulmonary artery endothelial cells [25].

The composition of UFP from diesel vehicle emissions is highly heterogeneous. It comprises of a complex mixture of chemical species, including black carbon (soot), metals and trace elements, as well as aromatic hydrocarbons and heterocyclic organic compounds, etc [26]. Certain chemical species such as organic carbon (OC), low molecular weight Polycyclic Aromatic Hydrocarbons (PAHs) and trace elements reportedly contribute to the toxicity of PM emitted from different vehicles [27] while the organic and metal PM components account for the pro-inflammatory effects [28-30]. Toxicity studies indicate that PM chemical composition is an important metric in assessing the effects of PM exposure [31].

In this study, we assessed the pro-inflammatory effects of UFP emitted from a diesel truck that was operated under two different driving cycles, i.e., idling mode (UFP1) versus urban dynamometer driving schedule (UDDS) (UFP2) on human aortic endothelial cells (HAEC). UFP2 contained a higher level of redox active metals and organic compounds compared to UFP1 on a per PM mass basis. Both UFP1 and UFP2 induced oxidative stress in HAEC. However, only UFP2 induced pro-inflammatory responses via NF-κB signaling. Our data suggest that even for the same diesel vehicle, different driving cycles result in PM emissions comprising different chemical components, thereby contributing to differential NF-κB-mediated pro-inflammatory responses.

## Results

### Chemical components of UFP collected from different driving cycles

**Figure 1:**
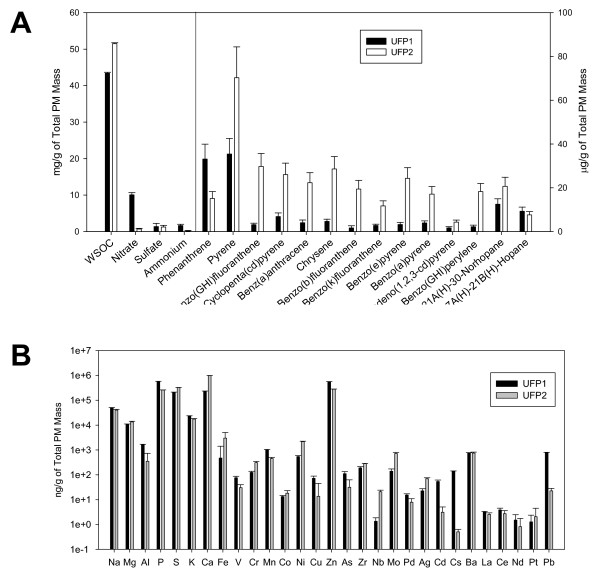
**Chemical compositions of UFP1 and UFP2**: **(A) **Comparison of PM bulk chemical species and speciated organic compounds ratios in total mass of UFP1 and UFP2. **(B) **Comparison of metals and trace elements ratios in total mass of UFP1 and UFP2.

The UFP collected from the tested diesel truck under the idling mode (UFP1) versus the urban dynamometer driving schedule (UDDS) (UFP2) revealed distinct chemical profiles on a per PM mass basis. Fig. 1A itemizes the individual ratios of bulk chemical contents normalized to total PM mass; namely, water soluble organic carbon (WSOC) and inorganic ions (Nitrate, Sulfate and Ammonium), as well as organic compounds, including Polycyclic Aromatic Hydrocarbons (PAHs) and hopanes. WSOC in the total mass of UFP2 was moderately higher than that in UFP1 at a ratio of 1.2 ± 0.1. The mass fractions of PAHs species were consistently higher in UFP2, except for phenanthrene, while the mass fractions of hopanes were comparable between the two UFP groups. Fig. 1B itemizes the ratios of the measured metals and trace elements normalized to total PM mass for UFP1 and UFP2. Calcium, Zinc, Phosphorous and Sulfur were the most abundant species in both UFP groups. The sum of the measured metals and trace elements fractions in UFP2 is moderately higher compared to UFP1 at a ratio of 1.2 ± 0.1. Of particular interest was the 4.3 ± 1.9-fold higher average mass ratio of the redox active metals, such as Iron, Chromium and Nickel, in UFP2 compared to UFP1. Detailed ratios of chemical components normalized to total PM mass can be found in the Additional file 1.

### UFP1 and UFP2 stimulated superoxide production and oxidative stress response genes

**Figure 2:**
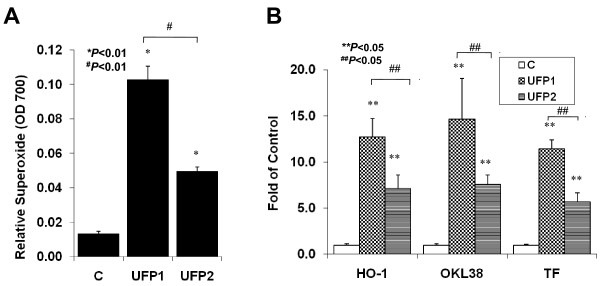
**UFP1 and UFP2 induced oxidative stress in HAEC**. **(A) **UFP1 and UFP2 similarly stimulated intracellular superoxide production. HAEC were treated with 50 μg/ml (15.6 μg/cm^2^) of UFP1 or UFP2 for 1 hour in the presence of NBT. Superoxide production was measured as absorbance at 700 nm. (B) Both UFP1 and UFP2 stimulated the expression of oxidative stress response genes. HAEC were treated for 4 hours with 50 ug/ml of UFP1 or UFP2. The expression of stress response gene HO-1, OKL38 and TF mRNA was measured by qRT- PCR. (C = control; * versus control, n = 3, *P *< 0.01; ** versus control, n = 3, *P *< 0.05; # UFP2 versus UFP1, n = 3, P < 0.01; ## UFP2 versus UFP1, n = 3, P < 0.05)

UFP emitted from the diesel vehicle stimulated oxidative stress responses in endothelial cells [20]. We herein investigated the vascular oxidative effects in HAEC exposed to UFP1 and UFP2. Both UFP1 and UFP2 induced endothelial cell superoxide production (C = 0.013 ± 0.0015, UFP1 = 0.103 ± 0.0081, UFP2 = 0.05 ± 0.0025, n = 3, *P < 0.01) (Fig. 2A). Consistent with our previous report [20], both UFP1 and UFP2 up-regulated oxidative stress response genes such as HO-1 and OKL38 as well as tissue factor (TF) (Fig. 2B, n = 3, ** *P *< 0.05). Nonetheless, the higher average mass ratio of the redox active metals, such as Iron, Chromium and Nickel, in UFP2 compared to UFP1, UFP1 appeared to be a stronger inducer of vascular oxidative stress (n = 3, ^##^*P *< 0.05).

### UFP2, but not UFP1, up-regulated pro-inflammatory gene expression

**Figure 3:**
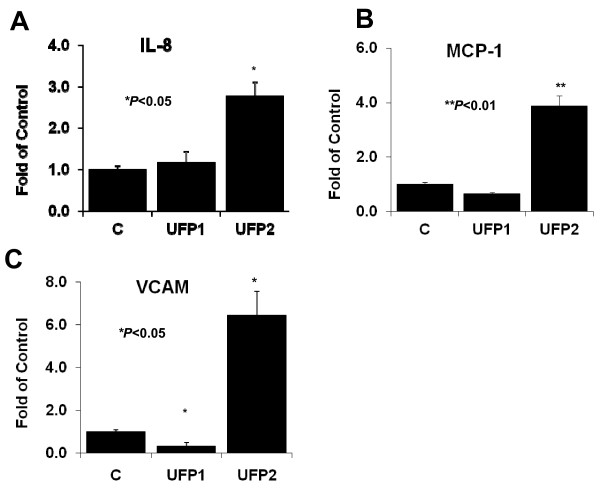
**UFP2 but not UFP1 stimulated the expression of inflammatory genes**. HAEC were treated for 4 hours with 50 μg/ml (5.2 μg/cm^2^) of UFP1 or UFP2. The expression of inflammatory chemokine gene IL-8 **(A) **and MCP-1 **(B) **and adhesion molecule VCAM **(C) **was measured by qRT- PCR. (C = control; * versus control, n = 3, *P *< 0.05, ** versus control, P < 0.01)

UFP2 at 50 μg/ml increased the expression of pro-inflammatory chemokine IL-8 and MCP-1 by 2.8 ± 0.3-fold and 3.9 ± 0.4-fold, respectively, as compared to control (n = 3, * *P *< 0.05, **P < 0.01) (Fig. 3A and 3B). UFP2 also up-regulated the expression of adhesion molecule VCAM by 6.5 ± 1.1-fold (n = 3, *P < 0.05), whereas UFP1 down-regulated VCAM expression (n = 3, *P < 0.05) (Fig. 3C). These data suggest UFP2 but not UFP1 induced significant pro-inflammatory responses in HAEC.

### Dose and time courses of UFP1 and UFP2 on gene expression in endothelial cells

**Figure 4:**
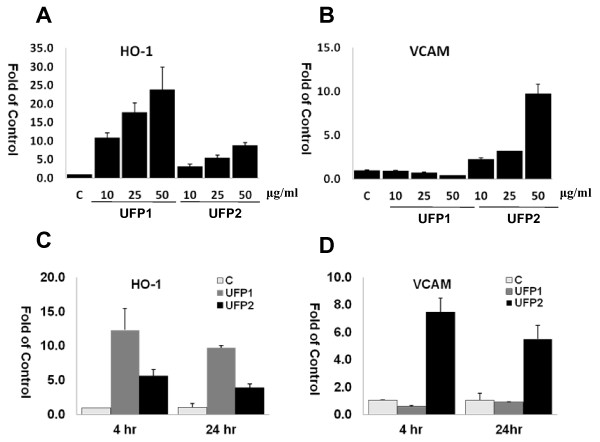
**Dose and time courses of UFP stimulated gene expression**. **(A) and (B): **HAEC were treated with indicated concentration of UFP1 or UFP2 for 4 hours. The expression of HO-1 **(A) **and VCAM **(B) **was measured by qRT- PCR. **(C) and (D): **HAEC were treated 50 μg/ml (5.2 μg/cm^2^) of UFP1 or UFP2 for 4 or 24 hours. The expression of HO-1 **(C) **and VCAM **(D) **was measured by qRT- PCR. (C = control; hr = hour)

To examine the dynamic effects of UFP on endothelial cells, we tested the dose and time course of gene expression in response to UFP1 and UFP2. We view HO-1 as representative of stress response genes and VCAM as representative of pro-inflammatory genes. Both UFP1 and UFP2 have similar dose effects on the expression of HO-1 (Fig. 4A). In contrast, UFP2 increased whereas UFP1 decreased VCAM expression in a dose dependently manner (Fig. 4B). Treatment of endothelial cells with UFP1 and UFP2 for 4 hours and 24 hours produced similar expression profiles for HO-1 and VCAM (Fig. 4C and Fig. 4D).

### Monocyte binding to UFP-exposed endothelial cells

**Figure 5:**
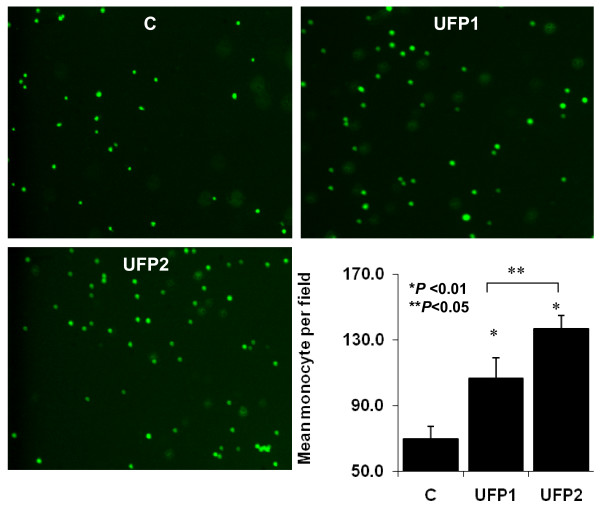
**UFP1 and UFP2 stimulated monocyte binding with different capacity**. HAEC grown to confluence were treated with 50 μg/ml (6.6 μg/cm^2^) for 5 hours. Monocyte binding assay was done with fluorescence-labeled THP-1 cells as described in methods. The averaged monocyte per high microscope field from 15 field of each condition was presented in the bar graph. (C = control; * versus control, n = 3, *P *< 0.01; ** UFP2 versus UFP1, n = 3, *P *< 0.05)

Pro-inflammatory oxidants such as oxidized low density lipoprotein (Ox-LDL) and oxidized phospholipids (Ox-PL) stimulate chemokine and adhesion molecule expression in vascular endothelial cells [32-34] that lead to increased monocyte binding to endothelial cells. Here, we performed fluorescence-labeled THP-1 monocyte binding assay to the confluent HAEC monolayer exposed to 50 μg/ml of UFP1 versus UFP2 for 5 hours. Both UFP1 and UFP2 stimulated monocyte binding to HAEC (n = 3, *P < 0.01) (Fig. 5). UFP2 induced a significantly higher number of monocyte binding compared to UFP1 (Control = 70 ± 7.5 monocytes per high power field, UFP1 = 106.7 ± 12.5, UFP2 = 137.0 ± 8.0) (n = 3, **P < 0.05) (Fig. 5). Analogous to Ox-LDL and Ox-LP, UFP induced pro-inflammatory effects.

### UFP2, but not UFP1, activated NF-κB signaling and NF-κB mediated the pro-inflammatory responses

**Figure 6:**
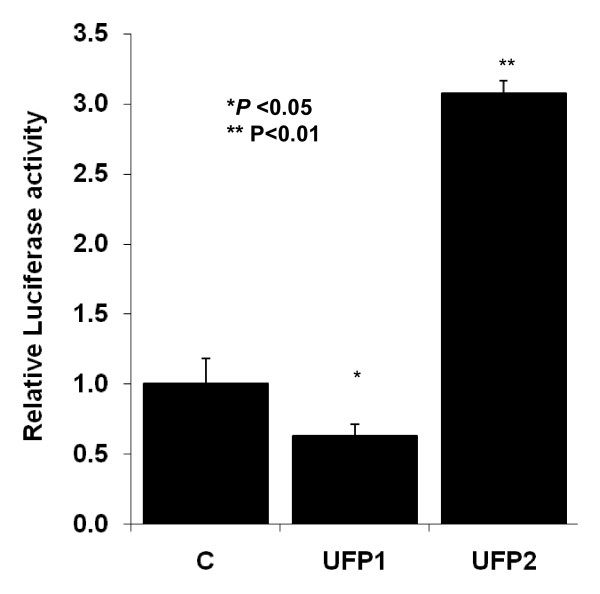
**UFP2 but not UFP1 activated the NF-κB signal transduction pathway**. HAEC grown to confluence were infected with Adv-LacZ and Adv-NF-κB-Luc overnight. The cells were then treated with 50 μg/ml (6.25 μg/cm^2^) of UFP1 or UFP2 for 24 hours. Cells were lysed and luciferase activities and β-Galactosidase activities were measured as described in methods. NF-κB reporter activities were normalized to LacZ levels and expressed as relative to control (C = control; * versus control, n = 3, *P *< 0.05; ** versus control, n = 3, *P *< 0.01)

NF-κB is a key signaling pathway mediating pro-inflammatory gene expression, including cytokines and adhesion molecules [35,36]. Using an adenovirus NF-κB Luciferase reporter (Adv-NF-κB-Luc), we demonstrated that UFP2 increased luciferase activities by 3.1 ± 0.1-fold compared to the control (n = 3, **P < 0.01), indicating the activation of NF-κB signaling pathway. In contrast, UFP1 decreased luciferase activities by 37% compared to control (n = 3, *P < 0.05) (Fig. 6).

**Figure 7:**
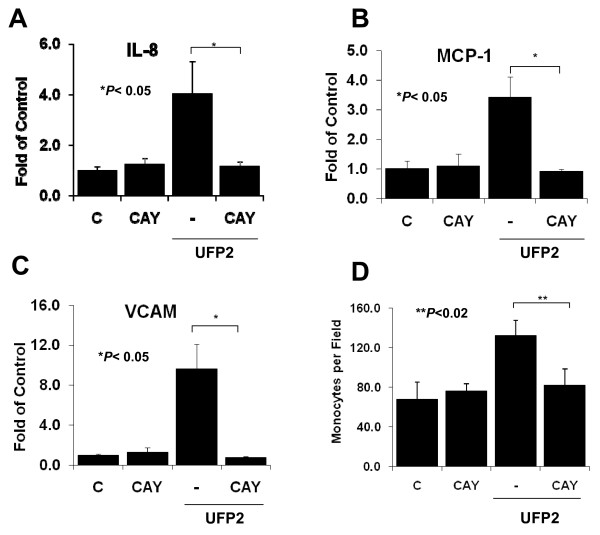
**NF-κB inhibitor CAY10512 blocked the pro-inflammatory effects of UFP2**. HAEC were pretreated with 2 μg/ml of NF-κB inhibitor CAY10512 for 1 hour followed by co-treatment with 50 μg/ml (5.2 μg/cm^2^) of UFP2 and CAY50512 for 4 hours (gene expression) or 5 hours (monocyte binding). The expression of inflammatory gene IL-8 **(A)**, MCP-1 **(B)**, and VCAM **(C) **was measured by qRT-PCR. Monocyte binding to HAEC was measured with fluorescence-labeled THP-1 **(D)**. (C = control; * n = 3, *P *< 0.05; ** n = 3, *P *< 0.02)

To assess if the activation of NF-κB pathway was implicated in UFP2-induced pro-inflammatory responses, we employed the NF-κB pathway inhibitor, CAY10512. UFP2-induced IL-8, MCP-1 and VCAM expressions were completely abrogated by CAY10512 (n = 3, *P < 0.05) (Fig. 7A, B, C). However, UFP-induced HO-1 expression was unaffected by CAY10512 (data not shown). CAY10512 also inhibited UFP2-stimulated monocyte binding to HAEC by 78% (n = 3, **P < 0.02) (Fig. 7D). Hence, our findings indicate that UFP2-induced pro-inflammatory activities were mediated via NF-κB signaling.

## Discussion

Ultra fine particles (UFP) emitted from diesel engines induced oxidative stress responses in endothelial cells [20,37]. In this study, we demonstrate that UFP emitted from an idling diesel truck (UFP1) versus the same truck under UDDS driving cycle (UFP2) induced differential levels of oxidative stress and pro-inflammatory responses in human aortic endothelial cells. UFP2 contained a higher level of water soluble organic carbon (WSOC) and consistently higher levels of organic compounds, including PAHs. Both UFP1 and UFP2 induced oxidative stress in HAEC. However, only UFP2 induced a pro-inflammatory response via NF-κB-mediated gene expression, and subsequently, monocyte binding to HAEC.

UFP1 and UFP2 had different levels of chemical composition; i.e., water soluble organic carbon and organic compounds on a per PM mass basis (Fig. 1). Water soluble organic carbon was reported to be closely associated with the consumption rate of dithiothreitol (DTT), a molecular assay that has been used as a surrogate measure for oxidative potential [38]. UFP2 contained a higher level of WSOC content (51.5 mg/g of PM) compared to UFP1 (43.4 mg/g of PM). However, our data suggested that UFP1 was a more potent inducer of oxidative stress than UFP2 in the context of lower organic carbon compounds (Figs. 2 and 3). Organic carbon refers to a complex mixture of organic compounds with various functional groups. Of particular interest are organic tracers of vehicle emissions such as PAHs, hopanes and steranes [1,2]. Li *et al*. [39] demonstrated that particle phase PAHs triggered oxidative stress in the cells via the formation of quinones. Hopanes and steranes are mostly found in lubricating oil and they are considered to be important biomarkers of fuel emissions [40,41]. Although the mechanisms by which these compounds cause health hazards are largely unknown, these organic species, along with other oil-derived components, have been reported to contribute to the inflammatory effects of inhaled emissions [42]. The per PM mass PAH content was consistently higher in UFP2 than in UFP1, and the average ratio (UFP2 to UFP1) was 5.5 ± 3.2, with the highest ratio of 11.7 observed for benzo (b) fluoranthene. Although our data suggest that higher level of organic compounds, such as WSOC and PAHs species in the total mass of UFP2 may account for its pro-inflammatory effects, additional studies need to be done to confirm the roles of individual compound or group of compounds.

Trace elements and metals are also important constituents of diesel engine emissions. While the sum of these species does not represent a substantial fraction of the PM mass, most of them are reported to promote free radical-based reactions in cellular or non-cellular bioassays [28]. As shown in Fig. 1B, Ca, Zn, P and S were the most dominant species in both UFP1 and UFP2. These elements are primarily ingredients of lubricant oil and sulfur containing fuels [43]. Higher fraction of metals and trace elements were found in UFP2 than in UFP1. These metal and trace element profiles displayed a different trend compared to organic compounds. The mass ratio of redox active transition metals (e.g. Iron, Chromium and Nickel) was 4.3 ± 1.9-fold higher in UFP2 than in UFP1. These metals may contribute to the pro-oxidant properties of UFP and up-regulation of pro-inflammatory gene expression [44]. Certain elements and metals have higher mass fractions in UFP1 compared to UFP2, notably Sodium, Aluminum, Phosphorous, Potassium, Manganese and Zinc. Mass fractions of Lead and Cadmium in UFP1 were 35-fold and 17-fold higher than in UFP2 respectively. Because of their ability to react with the function of thiol groups, the higher levels of Lead and Cadmium may be partially responsible for the higher oxidative stress induced by UFP1 than UFP2.

Oxidative stress is an emerging hypothesis to particulate matter (PM)-mediated cardiovascular diseases [45,46]. Exposure to UFP promoted atherosclerosis via systemic oxidative stress in ApoE-null mice [15]. In our study, both UFP1 and UFP2 induced a significant increase in superoxide production and anti-oxidant gene expression (Fig. 2), consistent with our previous report [20]. Exposure to different dose of PM is reported to induce hierarchical oxidative stress [47]; namely, a low dose exposure leads to antioxidant responses, whereas a high dose leads to a pro-inflammatory response. When HAEC were treated with the same dose of UFP1 and UFP2, only UFP2 stimulated the pro-inflammatory responses (Fig. 3 and Fig. 4,). However, UFP1 induced a higher level of oxidative stress response despite the lower average mass ratio of the redox active metals, such as Iron, Chromium and Nickel, in the total mass of UFP1 (Fig. 2). In this context, the hierarchical oxidative stress hypothesis alone may be insufficient to explain the differential effects induced by UFP1 and UFP2. Rather, the difference may also be due to the higher mass fractions of WSOC and PAHs species in the total mass of UFP2. The pro-inflammatory properties of UFP2 observed via NF-κB activation may be attributed to its enriched contents of organic species.

Pro-inflammatory oxidants such as Ox-LDL and Ox-PL, activate endothelial cells to express chemokines and adhesion molecules, and subsequently, monocyte recruitment to the endothelial cells [48]. Our study revealed that UFP, analogous to Ox-LDL and Ox-PL, also induced similar pro-inflammatory responses, suggesting a possible mechanism for the *in vivo *observation that UFP exposure promoted atherosclerosis in ApoE-null mice [15].

NF-κB is a major signal pathway that conveys pro-inflammatory responses to inflammatory stimuli such as TNF-α and endotoxin [35,36]. Some studies suggest that NF-κB is implicated in the pro-inflammatory effects of PM. For example, exposure to diesel exhaust particles (DEP) activated nuclear translocation of NF-κB in human bronchial epithelium[49]. Isolated rat capillaries exposed to DEP released TNF-α, a potent activator of NF-κB [37]. Our data demonstrated that UFP2, but not UFP1, activated the NF-κB signal pathway. UFP2 also induced inflammatory chemokines expression such as IL-8, MCP-1 and adhesion molecule such as VCAM. The NF-κB inhibitor, CAY10512, completely abrogated UFP2-induced pro-inflammatory gene expression, illustrating the important role of NF-κB signaling in response to UFP2. Dagher *et al*. reported that PM induced apoptosis of lung epithelial cells by TNF-α, a strong activator of NF-κB signaling [50]. Hartz *et al*. reported that DEP released TNF-α to bind to the TNF-α receptor, leading to P-glycoprotein up-regulation. However, inhibition of NF-κB did not block DEP-induced P-glycoprotein expression [37]. Therefore, the exact mechanisms by which UFP induce pro-inflammatory responses in the presence of specific chemical components warrant further investigations.

While both UFP1 and UFP2 stimulated monocyte binding to endothelial cells, UFP2 was a more potent inducer. UFP1 did not activate the NF-κB signal pathway, and CAY10512 did not completely inhibit UFP2-stimulated monocyte binding. These findings suggest that alternative mechanisms may contribute to UFP-stimulated monocyte binding.

## Conclusions

UFP emitted from a diesel truck running at the idle mode (UFP1) and UDDS driving cycle (UFP2) have distinctly different chemical compositions. These differences in PM chemical composition induce differential levels of oxidative stress and pro-inflammatory responses in human aortic endothelial cells. While both UFP1 and UFP2 induced oxidative stress in HAEC, only UFP2 induced a pro-inflammatory response via NF-κB-mediated gene expression, and subsequently, monocyte binding to HAEC.

## Methods

### Materials and Reagents

Endothelial cell culture media and reagents were obtained from Cell Application Inc. McCoy 5A media, fetal bovine serum (FBS), Calcein AM and other general cell culture reagents were obtained from Invitrogen Inc. CAY10512 was purchased from Cayman Chemicals Inc. Protease inhibitor (PI), phosphotase inhibitor cocktail and nitroblue tetrazolium were purchased from Sigma Inc. Real time PCR reagents were from Applied Biological Materials Inc.

### Preparation of Ultrafine Particles

The UFP used in the present study were collected from a 1998 Kenworth truck with 360,000 km in mileage. The truck was operated on two different driving cycles without any emission control device: idle mode and urban dynamometer driving schedule (UDDS). This experiment was part of a separate project to investigate the effectiveness of emission control technologies, developed to meet the 2007 and 2010 emission standards for heavy-duty diesel vehicles (HDDV) [51]. Experiments were carried out at the California Air Resource Board (CARB) heavy-duty diesel emission testing laboratory (HDETL) in downtown Los Angeles. Detailed dynamometer specifications and schematic particle collection set up were previously described by Biswas *et al*. [51].

A micro-orifice uniform deposited impactor (MOUDI) upstream of a nano-MOUDI (MOUDI-Nano MOUDI, MSP Corp., MN) was used to collect size-resolved PM samples (10-18 nm, 18-32 nm, 32- 56 nm, 56-100 nm, 100-180 nm, and 180 nm-2.5 μm). Each stage was loaded with pre-cleaned aluminum foil substrates. The MOUDI-Nano MOUDI was operated at a nominal flow rate of 10 lpm for multiple runs in order to accumulate sufficient mass for chemical analysis. A high volume sampler [52] operating at 450 lpm was employed to collect PM mass on Teflon coated glass fiber filters (20 × 25 cm) (Pallflex Fiberfilm T60A20-8x10, Pall Corp., East Hills, NY). We deployed the 47 mm Teflon filters (PTFE membrane filter, 2 μm, Pall Life Sciences, Ann Arbor, MI, USA) at 50 L min^-1 ^to collect particles denuded of volatile species downstream of the dilution channel. These substrates were subsequently used for the analysis of water-soluble metals and trace elements.

Gravimetric PM mass was determined by pre- and post-weighing the aluminum substrates from MOUDI-NanoMOUDI stages. The Teflon- coated glass fiber filters were then analyzed by the Shimadzu TOC-5000A liquid analyzer [53] for water soluble organic carbon (WSOC) and by means of ion chromatography (IC) technique for inorganic ions. A portion of these high volume samples was analyzed by means of gas chromatography-mass spectrometry (GC/MS) for organic compounds [54]. Water-soluble metals and elements were determined by means of Inductively Coupled Plasma - Mass Spectroscopy (ICP-MS) on 47 mm Teflon filters [55].

The remaining portion of the high volume samples was used to prepare the suspension of PM for the cell exposure tests. The filters were first soaked in the 10 ml of ultra-pure water (ultrapure Milli-Q deionized water; resistivity 18.2 megaohm; total organic compounds <10 ppb; particle-free; bacteria <1 colony forming unit/ml) for 30 minutes in endotoxin-free glass vial, followed by sonication for 30 minutes. After the particle suspension was transferred to endotoxin-free tube, another 10 ml of ultra-pure water was used to rinse the vials and repeat the aforementioned process. The chemical composition of the PM-bound water soluble organic carbon, inorganic ions and water soluble trace metals and elements in the aqueous suspensions were in good agreement with those measured on the original filters. Detailed extraction procedures have been described by Li, *et al*. [56]. Our control to UFP samples was made by extracting an identical blank filter with ultrapure Milli-Q, using the exact same procedures described for PM samples.

### Cell Culture

Human aortic endothelial cells (HAEC) (Cell Application) were cultured with endothelial cell growth media (Cell Application). The cells were used between passages 5 and 11. For UFP treatment, HAEC were incubated with 50 μg/ml of UFP in M199/0.1%FBS for specified time. For treatment with NF-κB inhibitor, CAY10512, cells were pretreated with 2 μg/ml of CAY10512 for 1 hour followed by co-treatment with UFP and inhibitor. Monocytic THP-1 cells were cultured in McCoy 5A supplemented with 10% FBS and 50 μM of β-mercaptoethanol.

### Superoxide Assay

Intro-cellular superoxide production was quantified using the nitroblue tetrazolium (NBT) assay as previously described [57]. Briefly, HAEC cells were plated in 96-well plate and were grown to confluent monolayer. The cells were washed with serum free M199 media and then treated with or without UFP in M199/0.1%FBS containing 0.2 mg/ml nitrobluetetrazolium (NBT). After one-hour treatment, the media were removed. The cells were then fixed and the extra cellular NBT was removed with methanol. After adding proper amount of KOH and DMSO, relative superoxide production was quantified in terms of optical density at 700 nm (O.D.700).

### Quantitative RT-PCR

Total RNA was isolated using the Bio-Rad kit following manufacturer's instruction. Potential genomic DNA contamination was removed with on-column DNase I digestion. 0.5-1 μg of total RNA was reverse transcribed with Bio-Rad's iScript cDNA synthesis kit. The expression of interested genes was analyzed at the mRNA levels using quantitative RT-PCR as previously described [20]. The expression of target genes was normalized to GAPDH. We also used 18S rRNA as control house-keep gene. Similar data were obtained. Primers used were as follows: MCP-1forward: GACACTTGCTGCTGGTGATTCTTC; MCP-1 reverse: TGCTCATAGCAGCCACCTTCATTC; IL-8 forward: ACCACACTGCGCCAACACAGAAAT; IL-8 reverse: TCCAGACAGAGCTCTCTTCCATCAGA; VCAM forward: TCGAACCCAAACAAAGGCAGAGTACGCA; VCAM reverse: AGGAAAGCCCTGGCTCAAGCATGTCATA;. HO-1 forward: GGCAGAGAATGCTGAGTTCATGAGGA; HO-1 reverse: ATAGATGTGGTACAGGGAGGCCATCA; TF forward: TTTGGAGTGGGAACCCAAACCCGTCA; TF reverse: ACCCGTGCCAAGTACGTCTGCTTCACAT; OKL38 forward: TCCTCTACGCCCGCCACTACAACATCC; OKL38 reverse: AGGTCCTGGAACACGGCCTGGCAGTCTTC.

### Reporter Gene Assay

HAEC were plated in 24 wells and grown to confluence. The cells were infected with Adenovirus-NF-κB-Luc (from Vector Biolabs) and Adenovirus-Lac Z (used as internal normalization control) at MOI of 1:100 overnight. The cells were then treated with or without 50 μg/ml of UFP (6.25 μg/cm^2^) in M199/0.1%FBS for 24 hours. Next day, media was removed and the cells were lysed in 100 μl of Reporter Lysis Buffer (Promega). Luciferase activities were quantified with TopCount NXT HTS (Packard) using Bright-Glow substrate (Promega). β-Galactosidase(encoded by Lac Z gene) activity was measured using β-Galactosidase reporter gene activity detection kit (Sigma) following the manufacturer's instruction. Reporter luciferase activities were normalized to Lac Z levels by β-Galactosidase activities and expressed as fold relative to control.

### Monocyte Binding Assay

#### a) Labeling human monocyte THP-1 with fluorescent dye

THP-1 cells, grown in suspension, were centrifuged, rinsed with phosphate-buffered saline (PBS) twice, and labeled with 2.5 μM Calcein AM (Invitrogen) according to the manufacturer's instruction. Fluorescence labeled THP-1 cells were centrifuged, rinsed with PBS twice, and re-suspended at 2.5 million/ml in HBSS/0.1%FBS.

#### b) Performing monocyte binding assay with fluorescence-labeled THP-1

HAEC were plated in gelatin coated 12 well plates and grown to confluence. The cells were treated with or without 50 μg/ml (6.6 μg/cm^2^) of UFP for 5 hours (triplicates). After removing treatment media, 400 μl of THP-1 cells labeled fluorescent Calcein dye were added into each well and incubated for 60 minutes in a humidified incubator with 5% CO_2 _at 37 degree C. Unbound THP-1 cells were removed by rinsing the wells three times with PBS. 0.2 ml of PBS/2%PFA was then added and incubated for 30 minutes at room temperature. The wells were then rinsed with PBS and 0.5 ml of PBS was added into each well. THP-1 cells attached to endothelial cells were counted under fluorescence microscope. Bound THP-1 cells were counted in five fields for each well and the averaged bound cells in each field were used for analysis.

For monocyte binding assay with NF-κB inhibitor CAY10512, HAEC were pre-treated with 2 μg/ml of CAY10512 for one hour and followed by co-treatment with 50 μg/ml (6.6 μg/cm^2^) of UFP and 2 μg/ml of CAY10512 for 5 hours.

### Statistical Analysis

All experiments were performed for three or more trials. Data were expressed as mean ± standard deviation (SD). For comparisons between two groups, student t-test was used for significance analysis. For comparisons among multiple values one-way analysis of variance (ANOVA) was used. A *P *value less than 0.05 was considered statistically significant.

## List of Abbreviations

UFP: ultra fine particles; PM: particulate matters; PAH: Polycyclic Aromatic Hydrocarbons; UDDS: urban dynamometer driving schedule; HAEC: human aortic endothelial cells; HO-1: hemooxygenase-1; TF: tissue factor; NBT: nitroblue tetrazolium; qRT-PCR: quantitative reverse transcription-polymerase chain reaction; DEP: diesel extracted particle; Ox-LDL: oxidized low density lipoprotein; Ox-PL: oxidized phospholipids; JNK: c-Jun N-terminal Kinases; NF-κB: nuclear factor kappa B; TNF-α: Tumor necrosis factor alpha.

## Competing interests

The authors declare that they have no competing interests.

## Authors' contributions

RL designed and performed the experiments, and wrote the manuscript. ZN prepared UFP samples and drafted part of the manuscript. RM did monocyte binding assays. JC did RNA isolation/cDNA synthesis. WT helped in experimental design and data interpretation. NJ helped in manuscript. TH and CS are the project leaders and critically revised the original and revised versions of the manuscript. All authors read and approved the final manuscript.

## Supplementary Material

Additional file 1 The chemical composition of UFP1 and UFP2 in total PM mass. Click here for file
