# Supplementary figures and images for: Ultrafine particles from diesel vehicle emissions at different driving cycles induce differential vascular pro-inflammatory responses: Implication of chemical components and NF-κB signaling
Source: Part Fibre Toxicol. 2010 Mar 22;7:6. doi: 10.1186/1743-8977-7-6 (PMC2859401; doi:10.1186/1743-8977-7-6)

Additional file 1: The chemical composition of UFP1 and UFP2 in total PM mass
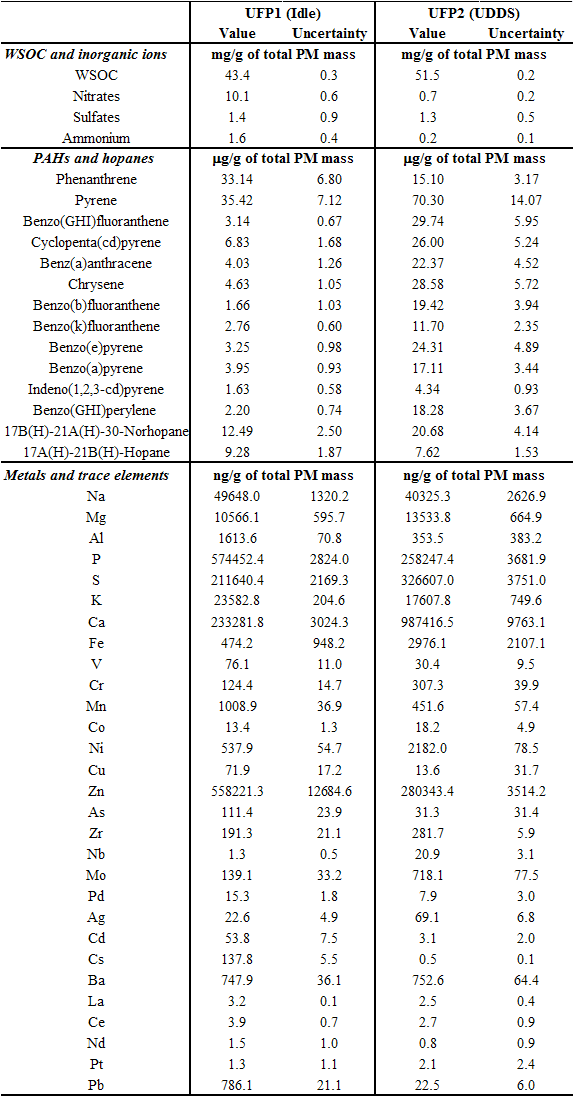

Supplement: Additional file 1 — The chemical composition of UFP1 and UFP2 in total PM mass. [file 1743-8977-7-6-S1.DOC]
